# A Case of Placenta Accreta Infection Managed by Hysterectomy After Treatment for Retained Products of Conception With Uterine Artery Embolization and Hysteroscopy

**DOI:** 10.7759/cureus.37854

**Published:** 2023-04-19

**Authors:** Hinata Fukuoka, Hirokazu Sato, Motonari Okabe, Kazue Togashi, Noriaki Oyama

**Affiliations:** 1 Obstetrics and Gynecology, Akita Red Cross Hospital, Akita, JPN

**Keywords:** placenta accreta spectrum, hysterectomy, uterine artery embolization (uae), surgical hysteroscopy, retained product of conception

## Abstract

Retained products of conception (RPOC) are frequently associated with previous cesarean section (C-section), abortion, and intrauterine operations, which may affect subsequent pregnancies. A 38-year-old female had a history of C-section and two abortions. After the second abortion, she underwent evacuation of RPOC and was treated with uterine artery embolization (UAE) and hysteroscopic resection. She became pregnant again and vaginally delivered an infant at full term. After delivery, RPOC was suspected on magnetic resonance imaging (MRI), but the patient was discharged for follow-up. She was rehospitalized with a diagnosis of infection and a placental remnant. Antibiotics did not improve the infection; therefore, she underwent a total hysterectomy. After the operation, signs of infection rapidly improved. The pathological diagnosis was placenta accreta. This case was considered a high-risk group for RPOC. In such rare and complicated cases, it is important to consider the possibility of recurrent RPOC and provide sufficient explanations before delivery for subsequent intensive management.

## Introduction

Retained products of conception (RPOC) depict a condition where pregnancy tissues, such as chorionic tissues, remain in the uterine cavity after delivery or abortion. They represent causes of postpartum hemorrhage and intrauterine infection, occurring in 1% of all deliveries [1]. The previous risk factors for RPOC include placenta accreta spectrum (PAS), a history of uterine surgery, and uterine artery embolization (UAE). Furthermore, the previously reported treatments include observation, intrauterine perfusion, UAE, hysteroscopic surgery, and hysterectomy, which are occasionally combined. The pregnancy rate 12 months after treatment is as high as 83.5% [2], but various complications may occur. Herein, we present a case of recurrent RPOC managed with a hysterectomy. We consider this a case in which most risk factors for RPOC are present: cesarean section (C-section), suction curettage, UAE, and hysteroscopy.

## Case presentation

**Figure 1:**
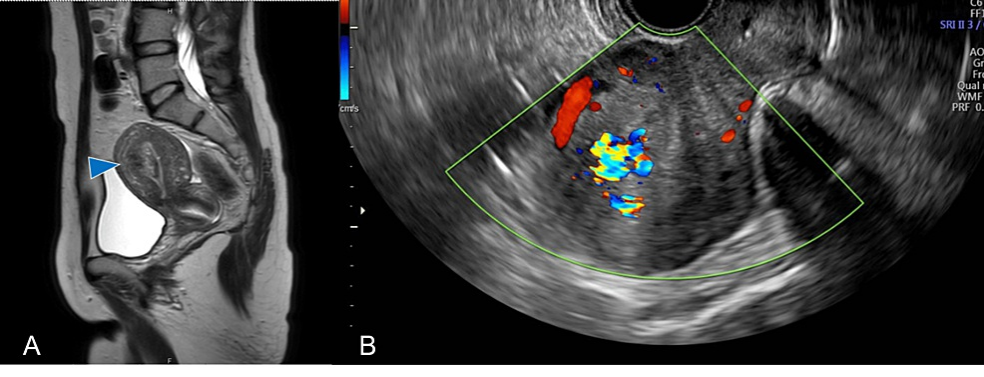
MRI and ultrasound for the diagnosis of RPOC A: Magnetic resonance imaging (MRI) showing a mass measuring 20×16×27 mm in size (arrows) protruding into the front wall of the endometrial cavity on a sagittal T2-weighted image. B: RPOC with hypervascularity in the intrauterine region. MRI: magnetic resonance imaging, RPOC: retained products of conception

A 38-year-old female with a history of C-section owing to cephalopelvic disproportion had two miscarriages in one year, five years after the C-section. The first miscarriage occurred at seven weeks of gestation, for which she underwent suction curettage. The second one was a spontaneous miscarriage at eight weeks of gestation, which resulted in her visiting our emergency room because of massive bleeding. Ultrasonography revealed placental remnants with abundant blood flow in the uterus; magnetic resonance imaging (MRI) to consider the treatment also revealed a tissue mass (size, 20×16×27 mm) in the uterine cavity, and RPOC was diagnosed (Figure 1).

**Figure 2:**
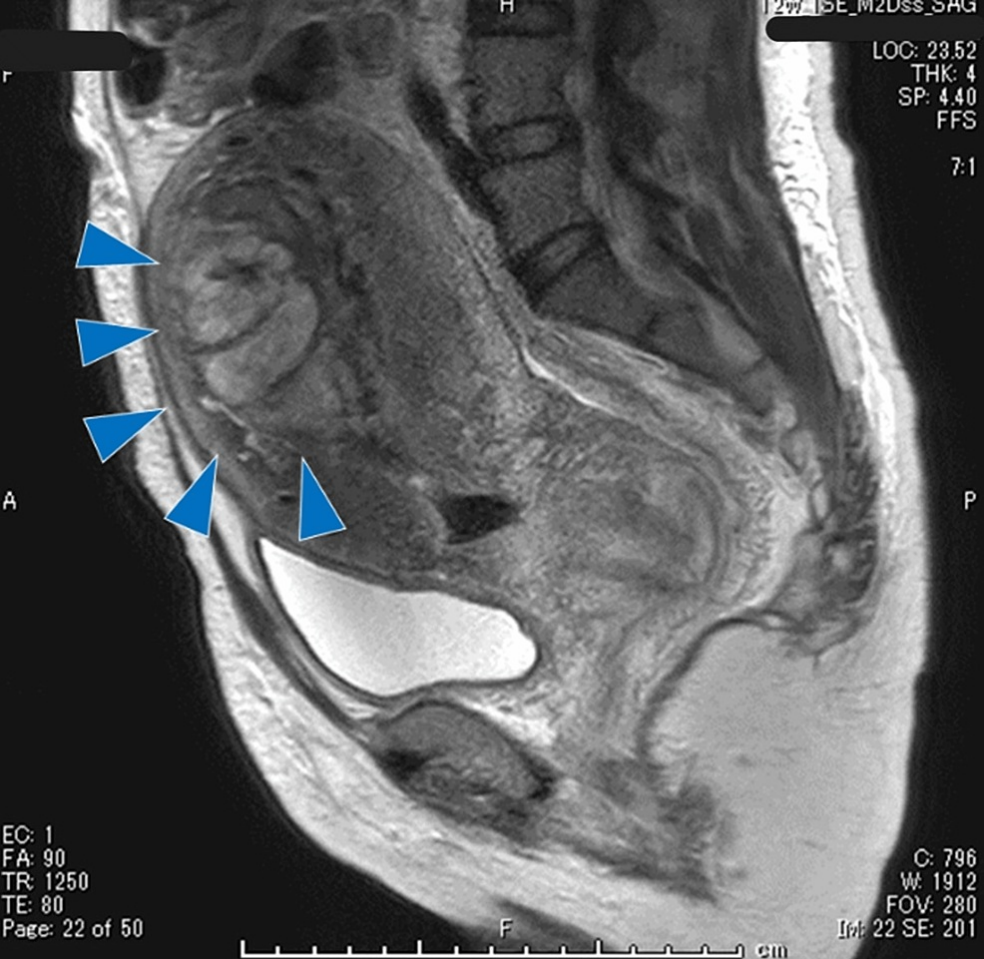
MRI for the diagnosis of placenta accreta MRI indicating a heterogeneous mass protruding into the endometrial cavity with high- and low-signal intensity on the T2-weighted image. The mass, 55×45×75 mm in size (arrows), was located from the bottom to the corpus of the uterine cavity. MRI: magnetic resonance imaging

Intrauterine suction evacuation or hysteroscopic resection after UAE was proposed as a treatment, the latter of which was chosen; thus, hysteroscopic resection was performed on the day after bilateral UAE. The residual placenta degenerated on the ventral side of the fundus of the uterus, and a small amount of bleeding occurred at the resection. The patient was discharged on the second day of hospitalization. Ensuing pathological examination revealed villous tissue and trophoblast cells scattered in the uterine muscle layer, which was diagnosed as placenta accreta. She was allowed to become pregnant six months after treatment, soon after which she became pregnant. The course of pregnancy was uncomplicated, and PAS was not suspected on ultrasound. She vaginally delivered a female infant at 40 weeks of gestation. The amount of blood loss during delivery was 2,750 mL, and half of the placenta was difficult to deliver. Bleeding from the uterine stopped, so we filled up iodoform dressing in the vagina and observed, and we decided to consider additional treatment. Because her hemoglobin (Hb) on day 2 postpartum was 4.8 g/dL, she needed a blood transfusion. MRI examination on the third day of puerperium helped diagnose retained placenta owing to placenta accreta rather than placenta percreta (Figure 2).

**Figure 3:**
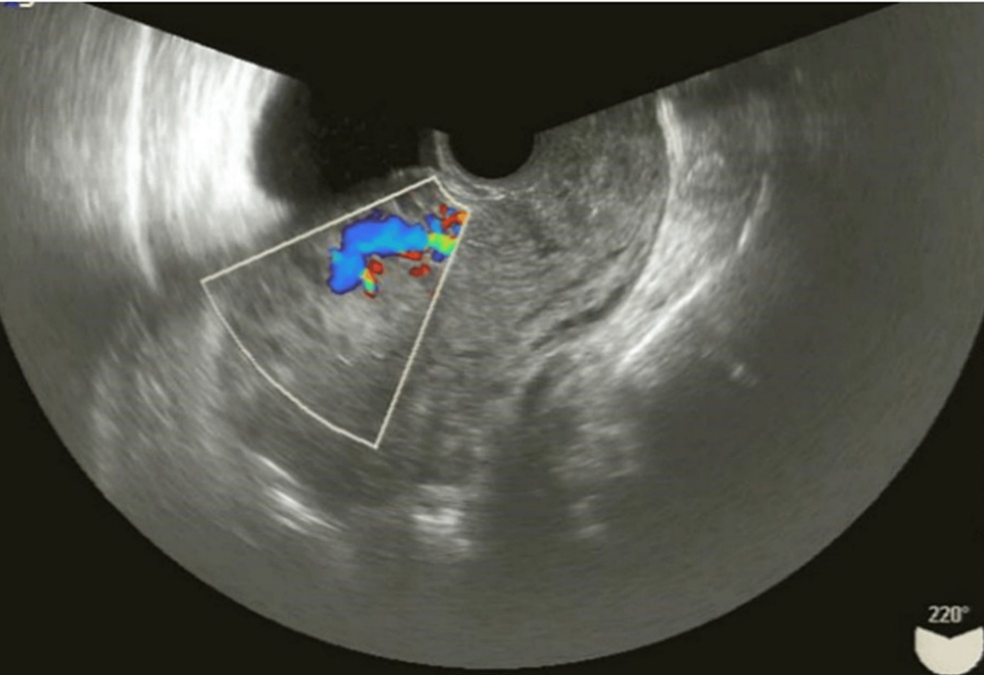
Ultrasound image of placenta accreta Ultrasound scan showing a hyper-echogenic intracavitary lesion measuring 78×36 mm, which appears highly vascular on color Doppler flow examination, consistent with RPOC. RPOC: retained products of conception

Transvaginal ultrasonography showed no blood flow in the placental remnants and no continuous bleeding; therefore, we expected spontaneous delivery of the residual placenta and decided to follow up during the next visit, a week after delivery. Subsequently, a small amount of bleeding and placental tissue delivery continued to occur daily. On day 17 postpartum, she visited our outpatient department with a fever and was prescribed oral antibiotics; however, the fever still continued. Doppler ultrasonography revealed a mass with blood flow in the uterus (Figure 3).

Two days later, she visited our hospital again because of high fever and was hospitalized with a diagnosis of residual placental infection. Although intravenous administration of antibiotics was initiated, elevated levels of C-reactive protein (CRP) and fever persisted. *Escherichia coli* was detected in the blood culture at the time of admission. We considered that conservative treatment would be insufficient to cure this condition. As a result of the consultation, the patient consented to undergo a total abdominal open hysterectomy. We performed a total abdominal hysterectomy, and the fever was rapidly alleviated on the second day postoperatively, and the inflammatory findings in the laboratory data showed improvement. The patient was discharged on the eighth day postoperatively, and the subsequent course was uneventful. Macroscopically, a residual placenta was found in the uterine cavity; the placental tissues adhered to the surface of the myometrium, and placenta accreta was pathologically diagnosed.

## Discussion

PAS, the cause of RPOC in this case, is a condition in which the villi invade the uterine myometrium, and part or all of the placentae adheres to the uterine wall, thereby preventing normal placental separation after delivery. The prevalence of PAS has been increasing yearly and is currently reported to be between 0.13% and 0.18% [3]. Placenta previa is the most common risk factor for PAS; other risks include a medical history of uterine surgical management (e.g., intrauterine curettage and myomectomy), previous C-section, and UAE [4]. Miller et al. [5] reported a considerably low (0.001%) probability of developing PAS in non-high-risk pregnant women. They also suggested that the incidence of PAS in cases with a history of C-sections increased as the number of C-sections increased: 14% for one cesarean section, 23% for two, 35% for three, and 50% for four. Therefore, uterine surgery, including C-section, is associated with a high probability of causing PAS, which subsequently develops into RPOC and would require therapeutic intervention. Among the various treatments described earlier, suction curettage and hysteroscopic surgery are combined as intrauterine operations and are frequently used for RPOC. As detailed in Table 1, intrauterine curettage is a simple but blind procedure of removing the remnant with a 40%-60% risk of bleeding and intrauterine adhesions [1].

**Table 1:** Advantages and disadvantages of each treatment RPOC: retained products of conception

	Advantages	Disadvantages
Suction and curettage	Handy, low cost	Blind procedure, so there is a risk of endometrial damage; risk of intrauterine adhesion
Hysteroscopy	Able to undergo direct observation and stopping bleeding, shorter mean time to subsequent conception, low risk of intrauterine adhesion	Hard to excise RPOC if the size is too large

Conversely, hysteroscopic surgery is an endoscopy-guided surgical procedure, which facilitates the excision of the remnant locally while arresting the bleeding by electrocoagulation. The amount of intraoperative bleeding is also minimal in hysteroscopic surgery; however, the rate of intrauterine adhesion after surgery is 7%-10%, which is lower than that in intrauterine curettage [6,7]. Based on these characteristics, hysteroscopic surgery is generally preferred for the treatment of RPOC. Intrauterine instrumentation is an effective treatment for RPOC but is associated with some complications. In addition to postoperative bleeding, it is reportedly related to placental abnormalities, such as placenta accreta, repeated RPOC, and increased frequency of intrauterine adhesions. Furthermore, Smorgick et al. [8] reported a significant difference in the risk of RPOC recurrence: 33% after intrauterine curettage and 12% after hysteroscopic surgery. The incidence of placental malposition was also significantly different, at 44% and 24%, respectively. Therefore, they concluded that hysteroscopic surgery had a lower overall risk than intrauterine curettage.

Although the treatment of choice should be considered according to the request of the patient, UAE is performed prophylactically at our hospital if RPOC with abundant blood flow is suspected on MRI or ultrasonography, and hysteroscopic surgery is performed after blocking the blood flow. In the present case, although the long diameter of the mass was small, about 2 cm, there was significant vascularity in the RPOC. Radiologists were consulted, and UAE and hysteroscopy were performed. The patient had a history of cesarean section and intrauterine procedures and was at risk for placental abnormalities and recurrent RPOC. We explained these potential complications and allowed her to conceive after RPOC treatment. Fortunately, she delivered the infant vaginally after treatment, but because this was the second RPOC and was complicated by an adherent placenta and bacteremia, we had to opt for a total hysterectomy. Considering the invasive nature of the procedure, laparoscopic surgery should have been chosen. However, since the patient was in the process of uterine restoration and intra-abdominal infection was suspected, we suggested that an abdominal total hysterectomy would be a better treatment. While the intrauterine procedure is an effective treatment for RPOC and can protect the possibility of pregnancy, it can also pose various risks to subsequent pregnancies. Therefore, in selecting and agreeing on a treatment for RPOC, it is imperative to present the expected complications in subsequent pregnancies.

## Conclusions

Despite the good progress of pregnancy after RPOC, it increases the complications from remnants of the placenta during the third stage of labor. In our hospital, we sometimes combine UAE and hysteroscopy for RPOC. Recently, hysteroscopy has been reported in most cases, and UAE is regarded as a safe and effective procedure to control blood flow. However, UAE might affect the endometrium in the future. Therefore, the advantages and disadvantages of treatment options should be considered. Investigating vascularity with color Doppler and undergoing UAE for cases of massive bleeding are crucial. In the case of various risk factors of pregnancy and deliveries, such as this case, we reconsider methods of examinations and timing of policy decisions and would like to make use of this experience in future cases.
